# Need for Better Blood Pressure Measurement in Developing Countries to Improve Prevention of Cardiovascular Disease

**DOI:** 10.2188/jea.JE20140146

**Published:** 2015-02-05

**Authors:** Pietro Amedeo Modesti, Eleonora Perruolo, Gianfranco Parati

**Affiliations:** 1 Department of Clinical and Experimental Medicine, University of Florence, Florence, Italy; 2 Department of Health Sciences, University of Milano-Bicocca; Department of Cardiology, S.Luca Hospital, IRCCS Istituto Auxologico Italiano, Milan, Italy

**Keywords:** non communicable disease, cardiovascular prevention, low income countries, global health, low resource settings

## Abstract

Hypertension is now the foremost cause of disability and is responsible for the highest percentage of attributable death among risk factors. These global changes are mainly due to the increase in the prevalence of hypertension in most low- and middle-income countries (LMICs) as a consequence of relevant socioeconomic changes occurring during the last decades. Implementation of global prevention efforts urgently needs to be accelerated because of the increasing incidence of haemorrhagic stroke, renal failure, and hypertensive heart disease in developing countries. Blood pressure (BP) measurement has different implications in epidemiological studies performed in low-resource settings. First, the frequency of blood pressure measurement is a simple but reliable indicator of access to healthcare in epidemiological studies, which may disclose the favourable effects of urbanization; the opportunity to have BP measured increases hypertension awareness, facilitates drug treatment, and leads to better achievement of BP control. Second, BP measurement is a key element in cardiovascular risk stratification, focusing solely on the preferred strategy in low-resource settings where costs of biochemical tests might be less sustainable. Third, the issue of obtaining reliable estimation of BP values is crucial to achieve sound data on the burden of hypertension in LMICs, and some aspects of BP measurement, such as the use of reliable automated devices, the number of measurements/visits to achieve a consistent diagnosis of hypertension, and the possible confounding effect of environmental factors, must be closely considered.

## HYPERTENSION AND EPIDEMIOLOGICAL TRANSITION

Hypertension is now the biggest contributor to the global burden of disease and to global mortality.^1^ In 2010, hypertension was responsible for the highest percentage of attributable death (13%)^1^^,^^2^ and was the foremost cause of disability,^3^^,^^4^ accounting for more than 20% of global disability-adjusted life years (DALYs) in adults aged 70 years and older and around 15% in those aged 50–69 years.^3^ Almost 1 billion people have uncontrolled blood pressure (BP) worldwide, with hypertension occurring in approximately 40% of people over age 25. Most importantly, the prevalence of hypertension is increasing, especially in low- and middle-income countries (LMIC).^3^^,^^5^

Between 1990 and 2008, the average systolic BP in Kenya increased from 127 to 132 mm Hg, whereas it was reduced by about 3 mm Hg in the United States.^6^ Epidemiological transition refers to the shift that occurs in developing countries as mortality rates from infectious diseases and nutritional deficiencies decrease and mortality rates from non-communicable diseases increases. The majority of LMICs have already passed through the first stage of epidemiological transition, characterized by high prevalence of infectious disease, to enter the second stage, marked by an increase in rates of diseases related to hypertension, such as haemorrhagic stroke, renal failure, and hypertensive heart disease.^2^^,^^7^ This shift or transition in disease and mortality rates reflects economic development, urbanization, industrialization, and changes in social organization within countries and regions, with increased exposure to risk factors driven by changes in diet, physical activity, and environment.

Implementation of global prevention efforts urgently needs to be accelerated, and overweight and obesity, along with increased salt and alcohol intake, are now recognised as important risk factors for hypertension.^8^^–^^13^ Prevention efforts for hypertension need to start by tackling nutrition and diet at an early age. Salt-reduction strategies are extremely important; 2 g/day of sodium (equivalent to 5 g/day of salt) is the value currently recommended by the World Health Organization,^14^ and by the recent joint guidelines from the European Society of Hypertension and European Society of Cardiology (class 1, grade A lifestyle modification).^15^ When sodium intake was <2 g/day, systolic BP was reduced by 3.47 mm Hg (95% confidence interval [CI], 0.76 to 6.18) and diastolic BP by 1.81 mm Hg (95% CI, 0.54 to 3.08) compared to intake of ≥2 g/day.^16^ Increased sodium intake was associated with an increased risk of stroke (risk ratio [RR] 1.24; 95% CI, 1.08 to 1.43), stroke mortality (RR 1.63; 95% CI 1.27 to 2.10), and coronary heart disease mortality (RR 1.32; 95% CI, 1.13 to 1.53).^16^ In rural areas, the majority of salt intake is derived from that added to food and during cooking by consumers,^17^ whereas in urban areas, where more than 50% of the world’s population now lives,^18^ the increased salt intake is mainly derived from consumption of processed food.^18^^,^^19^ Regulatory interventions to reduce salt consumption can hardly be undertaken by a single local health authority in LMIC, and an international approach targeting food production companies is required to ensure a reduction in salt content in processed foods. Population strategies for achieving a moderate reduction in population salt intake may vary according to the setting, relying more on education and health promotion in the less-developed areas of LMICs.^20^^–^^24^

The above-mentioned epidemiological transition is both a useful concept for understanding trends in disease burden in populations and an opportunity for health policy leaders to enact timely policy changes. For example, the comprehensive and sustained study of the epidemiology of stroke performed in China during the last few decades shed light on the relatively rapid socioeconomic changes that made hypertension an important problem in China. In the Sino-MONICA population,^25^ the first step in the transition was associated with increasing disease burden related to hypertension, such as haemorrhagic stroke, whereas ischemic heart disease and ischemic stroke emerged at later stages in the transition. Geographic differences in hypertension prevalence observed in China (27% in the Northern region vs. 12% in the South-western region)^26^ corresponded to geographical differences in the stroke incidence.^25^^,^^27^

These previous findings subsequently spurred the implementation of a high-level and sustained government commitment to the prevention and control of chronic, non-communicable diseases and their risk factors.^28^ This improvement in control of BP led to a 1.3% reduction in haemorrhagic stroke per year.^25^ Improvements in care at the hospital level have also led to reductions in fatality from haemorrhagic and ischemic stroke by 1.7% and 0.5% per year, respectively.^25^ However, with the progression of epidemiological transition, the prevalence of diabetes increased significantly in recent decades, reaching epidemic proportions in China.^29^ Recent data suggest that this epidemic might worsen further in the next few years, as over 40% of 18-to-29-year-olds are at risk of developing diabetes.^29^

As a result of policy reform, lifestyles in many parts of China have become more westernized, and these changes are more evident in urban than in rural areas. There are no current nationwide surveys on the prevalence of hypertension in China, and comparison between studies can be difficult because the number of BP measurements can influence the classification of patients as hypertensive. However, according to a recent meta-analysis comparing data from several previous national hypertension surveys published between January 2002 and June 2012, the prevalence of hypertension is now higher in urban areas than the Chinese national average.^30^ Subgroup studies also confirm the higher prevalence of hypertension in northern cities and among males.^30^ In 2011, the European Society of Hypertension established a Working Group on “cardiovascular risk in low-resource settings,” which brought together cardiologists, diabetologists, nephrologists, clinical trialists, epidemiologists, economists, and other stakeholders to investigate new strategies for cardiovascular (CV) risk assessment in population studies in LMICs and to assess future implications of these global changes for Europe.^31^ The growing rate of immigration might indeed influence the current favourable negative trend of CV mortality in Europe. Data regarding health and health needs of Chinese populations living in Europe are limited to a single study performed in the United Kingdom,^32^ although Chinese migration to Europe is mainly concentrated in relatively southerly countries (ie, Italy and Spain; Chinese now ranks third among the migrant communities living in Italy). Most importantly, only four European member states currently offer undocumented migrants access to primary care medicine for chronic diseases.^33^ Given the above, European countries might be missing potential opportunities to deal with migrant health, as history ignored might become history repeated, and a new wave of CV mortality could ensue in Europe in the coming decades.

## BP MEASUREMENT AS AN INDICATOR OF ACCESS TO HEALTHCARE

The growing rate of urbanization in LMICs makes a further increase in hypertension prevalence likely. However, the issue is quite complex, as urbanization may also offer favourable opportunities to reduce hypertension prevalence by improving rates of achieving high-level education and dissemination of knowledge of prevention, as well as by increasing access to health services.^34^^–^^36^ In urban areas, mass media (newspapers, magazines, radio, and television) may have a powerful influence on risk perception and may help foster public awareness of the need to reduce salt consumption.^37^ Media, including films, television shows, magazines, and more recently, the Internet, are the main sources of lifestyle influence around the world.^38^ Involvement of media is a recognized approach to change cultural models and encourage adoption of healthy lifestyle, especially among young people.^39^ New and highly interactive social networking sites are now popular with young people, and tools for measuring youth exposure to alcohol marketing in traditional media have been reported to be inadequate for social networking sites.^40^^–^^42^

Distance is an important obstacle to using health facilities in LMICs, where the rate of health services coverage for the population is often limited. Having BP measured in the preceding year in Yemen was found to be closely associated with hypertension awareness, drug treatment, and the possibility of achieving BP control.^43^ When moving from remote areas of the country to the capital area, having BP measured in the preceding year markedly increased.^43^ In the same study, these favourable effects of urbanization were particularly evident in women. After adjustment for age, urban/rural location, and education category, women were more frequently aware of their hypertensive status (OR 1.29; 95% CI, 1.01–1.64), were more likely to be treated with drugs (OR 1.31; 95% CI, 1.03–1.68), and achieved better BP control (OR 1.75; 95% CI, 1.15–2.67) than men.^44^ The favourable effect of urbanization on women with respect to hypertension control has also been consistently observed in different world regions.^45^

Reliable estimation of BP values is a cornerstone of CV risk stratification. At the patient level, the WHO NCD Research Agenda suggests that the delivery of low-cost multi-drug treatment through a primary healthcare approach should be based on CV risk.^46^^–^^48^ The WHO suggests performing CV risk stratification according to WHO/ISH risk prediction charts using a limited panel of data (age, gender, systolic BP, type 2 diabetes mellitus, smoking status, and total serum cholesterol).^46^^,^^49^ These charts were constructed using data obtained in the Comparative Risk Assessment Project^50^ and the Asia Pacific Cohort Studies Collaboration,^51^^–^^55^ in order to overcome limitations of previously used equations, which were derived from populations of European ancestry in high-income countries.^56^^–^^60^ WHO/ISH risk prediction charts may thus enable CV risk assessment and prediction in LMIC populations of all WHO sub-regions on the basis of age, BP, BMI, diabetes status, smoking habits, and cholesterol.^46^ The WHO proposed different 10-year total cardiovascular disease (CVD) risk thresholds for intensive intervention, which may be selected on the basis of local resources (20% for high-resource settings; 30% for medium-resource settings; and 40% for low-resource settings). As the CV risk threshold for drug treatment is lowered, there is a concomitant increase in health benefits.^47^ However, screening populations to identify those with very high risk (blood glucose and lipids assays) may be much more expensive than treating only hypertension. Indeed, in many low-resource settings, there are no facilities capable of even performing a cholesterol assay. To address this issue, an alternate version of the WHO/ISH charts was developed for use in cases where measurement of cholesterol level is not possible. If resources are limited, the preferred strategy is to only address hypertension and smoking, omitting assessment of lipid levels and diabetes status because of the lower risk attributed to those factors. Therefore, although CV risk stratification might be especially important for health systems of countries with very limited resources, screening to identify those with very high risk may be prohibitively expensive. The issue of obtaining reliable estimates of BP values in LMICs remains a bottleneck.

## BP MEASUREMENT IN LOW-RESOURCE SETTINGS

Three aspects of measurement may be relevant for ensuring reliable BP estimation at the patient (clinics) and population (surveys) levels in LMIC: the use of reliable automated devices, the number of measurements/visits to achieve a consistent diagnosis of hypertension, and the possible confounding effect of environmental factors (eg, temperature). New technologies for easier and more reliable BP measurements in LMICs may improve hypertension management in this context. In 2002, the WHO established a committee to develop technical specifications for an accurate and affordable BP measuring device for clinical use in low-resource countries.^61^ The objectives of the project were to discuss the preferred characteristics and to develop technical specifications for such a device. Identified barriers to accurate and affordable BP measurement in developing countries included the absence of accurate, easily-obtainable, inexpensive devices for BP measurement; marketing of non-validated BP measuring devices; high cost of available BP devices; limited awareness of the problems associated with conventional BP measurement techniques; and lack of trained manpower and limited training of personnel.^61^ To fulfil requirements related to BP measurement, the project concluded that a BP measuring device should be affordable (costing not more than €20) and extremely simple to use, but the device should also be accurate and robust, so that it could be easily used for repeated BP measurements. This initiative challenged manufacturers of BP measuring devices to produce an affordable, robust, and accurate tool. Out of several devices produced in this context and tested, one device—the Omron HEM-SOLAR—fulfilled the validation criteria of the International Protocol^62^ for systolic BP and also performed well in field-testing carried out in three centres in Uganda and Zambia, where healthcare workers preferred it to the mercury sphygmomanometer.^63^ To date, nearly all devices used in the research,^63^ which started in 2008, are still in good functional condition. This longevity is proof of their robustness. A recent comment in *The Lancet* emphasized that a major contributing factor to the rising tide of hypertension in African countries is the lack of a “simple, reliable, and accurate device for measuring BP”.^64^ Similar devices that are easy to use, inexpensive, accurate, robust, and that can be powered using photovoltaic cells (solar power) would prove valuable in improving the diagnosis and management of hypertension in many countries across the world.

In clinical settings, the diagnosis of hypertension requires recording BP measurements for several days over repeated visits.^65^^–^^67^ However, conventional estimates of hypertension prevalence in LMICs are often based on data collected at a single visit, so a potential bias exists when this information is used for cost estimations. The relevant logistic difficulties and personnel costs of a strategy based on two separate visits led the WHO STEPwise approach to Surveillance (STEPS) to adopt a single-visit strategy for assessing CV risk. The program allows structured between-countries comparison and within-country follow-up of the implementation of prevention strategies, although its results cannot be directly applied to cost estimation for prevention strategies. When the survey strategy included a second confirmatory visit (2-visit strategy), the prevalence of hypertension was 12% lower than when based on a single visit in a cohort of subjects aged 62 ± 11 years,^68^ 35% lower in a cohort of subjects aged 39 ± 9 years,^69^ and 35% lower in a nationwide survey of subjects aged 15 to 69 years.^70^ Contrary to expectations, misclassification was more common at young ages, with two-thirds of men <30 years of age having normal BP values at the second visit.^70^ Screening of subjects at high CV risk at the first visit is crucial. It is infeasible to perform an echocardiogram on all participants in a national survey; however, the inclusion in the test panel of a urine dipstick test for proteinuria might be a sustainable approach. Assessment of proteinuria allows classification of a patient in the high or very high class of 10-year risk of CV mortality, as defined by international guidelines for the management of hypertension.^65^^,^^66^^,^^71^^–^^73^ When urine dipstick testing for proteinuria was added to the parameters included in the STEPS approach in a national survey,^70^ the prevalence of subjects classified at high or very high CV risk at visit 1 remained stable at visit 2. More precisely, only 1.9% of subjects classified at high or very high CV risk at visit 1 moved to average, low, or moderate CV risk categories at the second visit.^70^ Therefore, a low-cost dipstick test for proteinuria may prove a useful tool for screening hypertensive subjects who are already at high risk at their first visit. Current strategy is to perform the dipstick test in subjects at increased risk of CKD (so called “selective screening”),^74^ and the WHO suggests performing a urine dipstick test only if SBP ≥ 140 or DBP ≥ 90 mm Hg, and the dipstick test for proteinuria is not included in the WHO STEP program.

Only minor attention is paid to three points regarding kidney disease that might be relevant to the context of LMICs. The first point is that we now have evidence that kidney damage hazards (death, CV death, and end-stage renal disease [ESRD]) may be independent of diabetes and hypertension.^75^^,^^76^ Data from Nepal, Bolivia,^77^ and Yemen^44^ showed that more than 5% of people aged younger than 60 years without previous history of diabetes and hypertension had microalbuminuria/proteinuria. The second point is that in China, where the prevalence of proteinuria is high,^78^ glomerulonephritis (mainly post-infectious) is a major cause of CKD.^79^ The third point is that, although the possibility of treating kidney failure may be limited in LMICs, evidence is emerging that CKD and CVD have a major impact on macroeconomic development, resulting from diminished labour supply due to premature death and disability in people of working age. Over the last decade, the number of those requiring dialysis increased annually by 6.1% in Canada, 11% in Japan, and 9% in Australia.^80^ These rates of increase are not sustainable in countries such as India and Pakistan where <10% of all patients with ESRD receive any form of renal replacement therapy.^80^^,^^81^ Considering that off-patent antihypertensive drugs, such as angiotensin-converting enzyme inhibitors, can reduce albuminuria and prevent decline in glomerular filtration rates,^82^ as well as reduce incidence of CV events,^83^^,^^84^ prevention programs could led to early identification and treatment of both hypertension and renal abnormalities, with the primary goal of reducing mortality and morbidity associated with CV and ESRD.

Guidelines on BP measurements recommend a standardized room temperature in hypertension clinics, since the inverse relationship between diastolic pressure and the temperature of the room in which measurements are made is well known.^85^^,^^86^ However, when BP measurements are made in comfortably warm rooms, a negative relationship between outdoor temperature and BP values was observed.^87^^–^^93^ In the French Three-City study, where the relationship between office BP (measured according to current guidelines) and outdoor temperatures (obtained from local meteorological offices) was prospectively investigated in 8801 participants over the age of 65, BP was decreased by 8.0 mm Hg at the lowest (<7.9°C) compared to the highest (21.2°C) temperatures. However, in contrast, average systolic BP was 5 mm Hg higher in winter than in summer.^93^ These relationships were independent from anthropometric data and baseline BP values but were related to subjects’ age. BP changes were more marked in subjects aged 80 years or older than in younger participants. The importance of seasonal BP variations is now considered in most clinical trials.^86^ Conversely, only one population study—a large survey performed in Yemen (HYDY)—specifically investigated the possible bias introduced by environmental temperature on hypertension burden assessment.^44^ According to the HYDY study,^43^ the odds ratio for hypertension diagnosis was 0.98 (95% CI, 0.96 to 0.99) per 1°C of temperature measured at home (logistic regression analyses adjusted for age, gender, education, and average air temperature at the two survey visits).

## CONCLUSIONS

The relative importance of dealing with high BP in LMICs has markedly increased over the last few decades, and high BP is now the leading risk factor for CVD. The establishment of a culture of prevention in LMICs requires the concerted, coordinated action of television, radio, newspapers, and magazines, with the support of international efforts, to limit salt content in processed foods. The adoption of a CV risk approach has relevant implications for patient care and healthcare resource allocation and decision making at regulatory levels. However, screening those with very high CV risk in LMICs may be much more expensive than treating only hypertension, and the importance of reliable estimation of BP levels is crucial at both the patient and population levels. The use and diffusion of low-cost, reliable automated devices is essential. However, assessment of hypertension burden on the basis of a single visit may lead to overestimation of prevalence and healthcare requirements, so implementation of appropriate survey strategies measuring the treatment gap on the basis of CV risk stratification (untreated individuals at high CV risk), rather than strategies based on individual risk factors (untreated hypertensive), have to be considered.
